# Investigating valley-dependent current generation due to asymmetric energy dispersion for charge-transfer from a quantum dot to single-walled carbon nanotube

**DOI:** 10.1038/s41598-023-30247-1

**Published:** 2023-02-22

**Authors:** J. Charoenpakdee, Ongart Suntijitrungruang, S. Boonchui

**Affiliations:** 1 Department of Physics, Faculty of Science, Kasetsart University, Bangkok, 10900 Thailand; 2 Center of Rubber and Polymer Materials in Agriculture and Industry (RPM), Faculty of Science, Kasetsart University, Bangkok, 10900 Thailand

**Keywords:** Single photons and quantum effects, Quantum optics

## Abstract

Single-wall carbon nanotubes (SWCNT), which consist of a two-dimensional hexagonal lattice of carbon atoms, possess unique mechanical, electrical, optical and thermal properties. SWCNT can be synthesized in diverse chiral indexes to determine certain attributes. This work theoretically investigates electron transport in different directions along SWCNT. The electron studied in this research transfers from the quantum dot that can possibly move to the right or left direction in SWCNT with different valley-dependent probability. These results show that valley polarized current is present. The valley current in the right and left directions has a composition of valley degrees of freedom where its components (K and K′) are not identical. Such a result can be traced theoretically by certain effects. That firstly is the curvature effect on SWCNT in which the hopping integral between $\pi$ electrons from the flat graphene is altered, and another is curvature-inducing $\sigma -\pi$ mixture. Due to these effects, the band structure of SWCNT is asymmetric in certain chiral indexes leading to the asymmetry of valley electron transport. Our results exhibit that the zigzag chiral indexes is the only type making electron transport symmetrical that is different to the result from the other chiral index types which are the armchair and chiral. This work also illustrates the characteristic of the electron wave function propagating from the initial point to the tip of the tube over time, and the current density of the probability in specific times. Additionally, our research simulates the result from the dipole interaction between the electron in QD and the tube that impacts the lifetime of the electron being in QD. The simulation portrays that more dipole interaction encourages the electron transfer to the tube, thereby shortening the lifetime. We as well suggest the reversed electron transfer from the tube to QD that the time duration of such transfer is much less than the opposite transfer owing to the different orbital of the electron’s states. Valley polarized current in SWCNTs may also be used in the development of energy storage devices such as batteries and supercapacitors. The performance and effectiveness of nanoscale devices, including transistors, solar cells, artificial antennas, quantum computers, and nano electronic circuits, must be improved in order to achieve a variety of benefits.

## Introduction

Photocurrent’s enhancement for solar cell modification, by quantum dot coupling to carbon nanotubes, is an emerging field with spectacular properties. The photoluminescence (PL) of semiconducting single-wall carbon nanotubes (SWCNTs) and multiwalled carbon nanotubes (MWCNTs) have been investigated in various physical properties such as the single-photon sources at room temperature^1–4^, the photogate effects caused by charge transfer^5,6^, etc. Regarding MWCNTs, the photoinduced current responses from nanocomposite coupling with MWCNTs was studied^7–9^ such as thiol derivative perylene compound and cadmium selenide quantum dot (QD). Formerly, scientists cannot synthesize single-wall carbon nanotubes (SWCNTs) that possess uniform electronic properties and particular chiralities, causing the major impediment for widespread applications. Nowadays, there is a growing interest to explore and synthesize specific well-defined carbon nanostructures, which include fullerenes, short nanotubes, and sidewalls of nanotubes, in order to control the synthesis of SWCNTs. Corannulene molecules used in the bottom-up synthesis of (5,5) SWCNT end-caps, for example, is a notable demonstration according to this manner^10–12^. As a result of these previous works with method’s development, SWCNT-based computing technology has been a recent interesting subject. Moreover, hybrid materials (as photo-electrodes) and the charge transfer from semiconductor nanoparticles (such as CdS, CdSe) as well as CdTe (donor) to SWNTs (acceptor), generating increased photon current, have been demonstrated by several groups. In regard to the charge transfer, there are diverse unexpected phenomena owing to this effect. Solution processed lead halide-based perovskite nanocrystals (PNCs), for instance, are very bright in luminescence but quenching in the presence of CNTs. This phenomenon is attributed to the electron transfer from PNCs to CNTs^7,13^. For the charge transfer, Functionalized Multi-walled Carbon Nanotube, added TiO$_2$ photoinduced interfacial hole transfer to carboxylic acid-functionalized multiwalled carbon nanotubes (MWCNT) from TiO$_2$, results in hole-doped MWCNTs and reduced TiO$_2$^14^. Nevertheless, concerning the effects of asymmetric energy dispersion on the charge transfer and photocurrent properties depending on chirality of SWCNT, there presently are no researches that consider these effects. Therefore in this research, we examine and consider these probable effects especially.

In theoretical discussion on the tight-binding model in SWCNTs, that takes into account the curvature’s effects, the formulation of the graphene sheet altered by the hopping integral between the $\pi$ orbitals and the curvature-induced $\sigma -\pi$ mixing on quantum transport of an electron in SWCNT^15^ is considered. Accordingly, certain properties from the curvature parameters arise. The results from this consideration have been presented and studied in various aspects. For example, due to the intrinsic magneto-optical properties of various SWCNT chiralities, it generates an asymmetry in the bright exciton recombination line, attributed to the free nature of excitons^16^. In addition, particular researches, both experimental and theoretical, elucidate the efficiency of absorption and emission of light by the individualized nanotubes relying on different chiralities^17,18^. In regard to the photoluminescence (PL) attributes of chiral SWCNT, certain works^19–21^ analyze its intensity by calculating photon-emission matrix elements for each chiral index (n,m). As shown by W. Izumida^22,23^, the effects of the two-dimensional Brillouin zone, in SWCNT, cause the electron’s transport asymmetric as a result of second-order perturbation in curvature-induced $\sigma -\pi$ mixing with Slater-Koster type projection in $\pi -\pi$ and $\sigma -\pi$ on the curved surface.

Recently, current-induced spin polarization and valley polarization were studied for semiconductor^24^ and hexagonal 2D materials phenomenons^25^. The current in such a material is generally composed of valley or spin degrees of freedom where its components are mostly not equal. Accordingly for the charge transfer effect on SWCNT, it generates the current referred to as valley-dependent current^26^. Its process entails a current generated in a transistor or other solid-state device by using the potential energy of the electron valleys in the device. Currently scientists successfully found a way to detect and separate the current of one specific valley and the other by observing Berry curvature with twisted light^26,27^. Our research is based on a fact that the difference in valley contribution to the currents can be used as a valley-dependent current. On Application, valley-dependent current has the potential to be used in the design of new types of transistors and other electronic devices. By utilizing the valley degree of freedom, it may be possible to create faster, more efficient devices that can operate at higher speeds with lower power consumption.

**Figure 1:**
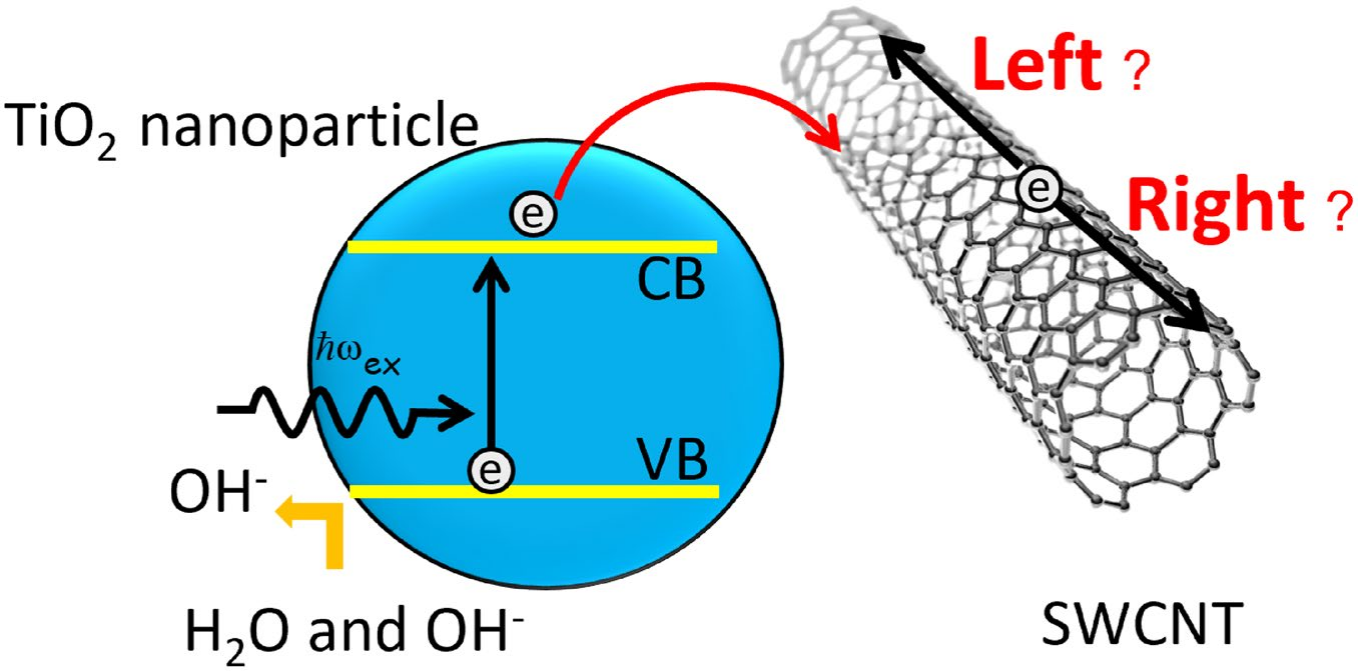
Schematic illustrations of interfacial contact between nanocarbon and TiO$_{2}$, and charge carrier movement in TiO$_{2}$-SWCNT.

This paper is organized as follows: we begin with the results and discussions from the simulation. The first is the probability of finding the electron transferred from QD at the specific location both in the left and right side. The second is the difference of arrival time of the electron propagating from the initial point to the certain position where we compare the arrival times between the left side with the right side. Third, we show the characteristic of wave function from the right going direction as well as the current density both in the left and right side at the particular times. The last result is the rate of electron transfer from QD to SWCNT with the probability to find the electron at QD, the left and right side over time in which we determine the interaction parameter differently. Then, we provide the conclusion and suggest some applications from our research. In the method section, we describe a theoretical discussion of the tight-binding model and asymmetric velocity for the chiral carbon nanotube. As well, we explain the investigation and mathematical detail of a photoinduced current event where the quantum dot absorbs a photon creating an electron-hole pair as Fig. 1. Our formulation takes the perturbation theory framework, and the most useful representation of this operator in our work is the Dyson series.

## Results and discussions

Firstly, we investigate a photoinduced current event that a quantum dot absorb photon creating electron-hole pair, represented by an excited state $|ex\rangle$. As such, the QD serves as a two-level quantum emitter consisting of the excited state, $|ex\rangle$ (creating electron-hole pair) and the ground state $|g\rangle$. After the photon absorption, the holes scavenged by the ethanol and the electron completely transfer to the conduction band of the *K* valleys, while the electron transfers from the quantum dot to SWCNT, generating the sensitized right and left-hand photocurrent in SWCNT, motivated as^28,29^. The studied system is schematically illustrated in Fig. 1. The probability of an electron moving from a quantum dot to an electron state is usually the same in the *K* and $K'$ valleys of the nanotube. After that, the electron transport should be based on the difference in velocity between electrons travelling to the left and right ($v^{(K)}_ R \ne v^{(K)}_L$) for the *K* valley (or $K'$ valleys). Under the time-reversal symmetry, which requires the symmetry between *K* and $K'$ valleys, $v^{(K)}_R = v^{(K')}_L$ and $v^{(K)}_L = v^{(K')}_R$, this can be understood as the electrons in the *K* and $K'$ valleys have opposite angular momentum. From such a characteristic, if we derive the probability and the current of electrons from one valley, then we can obtain propagation dynamics of the electron from the other valley through time-reversal symmetry. For this reason, we logically simulate the valley-dependent current generation from only the *K* valley. Certainly, when we consider the contribution from the *K* and $K'$ valleys, total current in the left and the right hand of SWCNT should be the same absolute value. However, these valleys can be represented by a binary pseudospin that behaves like a spin-1/2 system; the electrons in the *K* valley can be labelled as valley-pseudospin up, and the electrons in the $K'$ valley can be labelled as valley-pseudospin down. Therefore, the left and the right-hand current should store binary information or “valleytronic”. The valley currents in the right and left directions have a composition of valley degrees of freedom where its components (*K* and $K'$) are not similar. Until very recently, there has been experimental progress in detecting the degree of freedom in the valley-dependent current for prospective valleytronic devices. Thus, after the electron transfer from QD to SWCNT, we can possibly observe the difference of composition of valley currents by using these detection schemes as^27,30–32^ For example, the valley optical selection rules can be applied to measure valley polarization. The left- and right-circularly polarized light interact differently with the (*K* and $K'$) valley current^26,33^. Ho, presented the principle and detection scheme in the direct experimental demonstration measuring valley polarization in two-dimensional materials with second-harmonic spectroscopy^34,35^.

**Figure 2:**
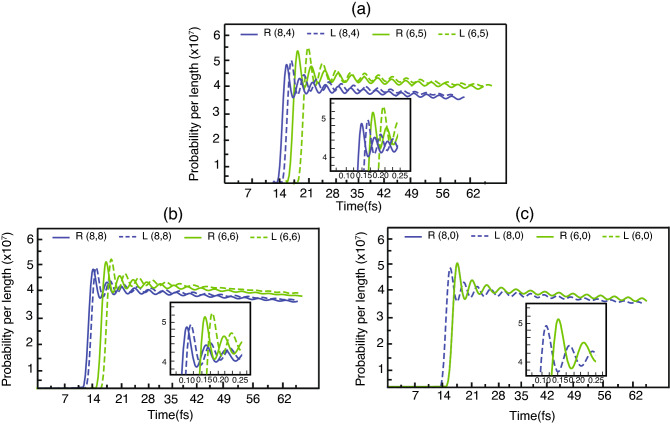
The probability of finding the electron transferred from quantum dot on the left direction (dot lines) and the right direction (solid lines) at the position $|\,z\,|= 40\, \text{nm}$ for the specific chiral indexs as (**a**) (8, 4) in blue lines and (6, 5) in green lines, (**b**) (8, 8) in blue lines and (6, 6) in green lines, and (**c**) (8, 0) in blue lines and (6, 0) in green lines. The inserts show the magnified lines (2 times) for each own graph in order to be observed vividly the difference of the peaks from left-going and right-going.

Based on our model, the transferred electron from the QD, at an initial state $(t = 0)$, is in the center of the tube. However, when the state evolves, the electron will transport from the center to the right or left tip of the tube. Figure 2 shows the probability for finding the electron at $z= -40\, \text{nm}$ (left hand side) and $z= 40\, \text{nm}$ (right hand side) per length in our system. Figure 2a–c belong to the different SWCNT types that are chiral, armchair and zigzag, respectively. Considering the zigzag type as shown in the inserted graph of Fig. 2c, the simulation demonstrates that the probabilities whether the right or left direction are the same. On the contrary, the chiral and armchair types (as simulated in the inserted graphs of Fig. 2a,b) illustrate that the probabilities from the left and right direction are different. These noticeable results can be ascribed by the band structure of each chiral type. The band structure of zigzag type is generally symmetrical whereas the band structure of chiral and armchair type is asymmetric^23^. Therefore, the asymmetry of the band structure is a primary reason why the probability between the right and left direction is different.

**Figure 3:**
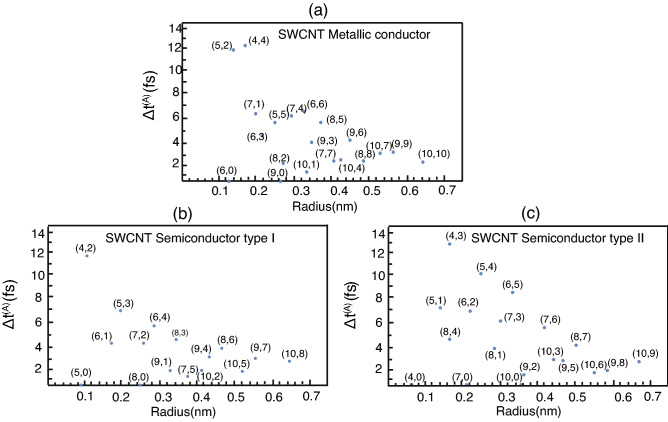
The differences of arrival time, depending on radius, are simulated from three types of SWCNT that are (**a**) metallic, (**b**) semiconductor type I and (**c**) semiconductor type II.

Considering Fig. 2a,b that are results from the asymmetry of the band structure, both figures portray that maximum probability for finding the electron at $|\,z\,|= 40\, \text{nm}$ (in the same chiral index) of the left direction is more than of the right direction. On the other hand, the electron of the right hand side arrive at $z= 40\, \text{nm}$ before the left side’s electron ($z= -40\, \text{nm}$). This result implies that the velocity of the electron in the right direction is more than that of the left direction. Regarding the different maximum probability, the result can be explained by the variable referred to as density of state that is inversely proportional to the velocity. In addition, from the Eq. (25), the probability is inversely proportional to the square of velocity. For this reason, the right direction’s maximum probability (where the electron’s velocity is more) is less than the left direction’s.

To investigate prudently, we calculate the arrival time $t^{(A)}_{L(R)}$ that is a global maximum point of probability density for finding the electron on the left and right-hand side as Eq. (31) for $z=-40$ nm and 40 nm respectively, denoted $max(P_{\eta }(\pm 40 \, \text{nm}, t^{(A)}_{L(R)}))\ge P_{\eta }(\pm 40 \; \text{nm}, t)$. Figure 3 shows the difference of arrival time defined as $\Delta t^{(A)}=t^{(A)}_{L}-t^{(A)}_{R}$ where Fig. 3a–c belong to the different types of SWCNT that are metallic, semiconductor type I and semiconductor type II, respectively. The result emphasizes that the difference of electron’s velocity between left and right direction (signified by the arrival time’s difference) depends on the chiral index of SWCNT despite the same SWCNT’s type. As well, the radiuses derived directly from Eq. (1) rely on the *n*, *m* of chiral index. According to our samples, the maximum arrival time’s difference is from chiral index (4,3) being semiconductor type II that is 12.8 fs approximately. Concerning the other types that are Metallic and Semiconductor type I, the maximum arrival time’s differences in our samples are 12.2 fs and 11.8 fs from the index (4,4) in Metallic and (4,2) in Semiconductor type I, respectively. Therefore, the results both from Figs. 2 and 3 confirm the certain effect leading to the different electron’s probability and velocity between right and left direction.

**Figure 4:**
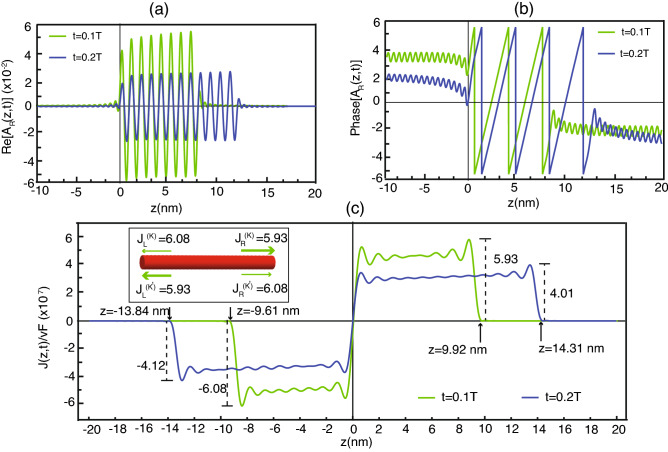
Show asymmetric wave function and current density for SWCNT (4,3) at the difference times 0.1 T for green line and 0.2 T for blue line where $T = 100 \; \text{fs}$ is the lifetime of the electron being in QD. (**a**) The real part of the right hand side wave function of electron along z-axis. (**b**) The phase of the right hand side wave function of electron along z-axis. (**c**) The total current density along z-axis.

To study the properties of electron transport thoroughly, Fig. 4a is a real part of the right hand side wave function, following Eq. (23) at the times 0.1 T and 0.2 T ($$T =100\,\text{fs}$$ is the lifetime of the electron being in QD) and Fig. 4b is the phase of this wave function. Figure 4c is the current density per fermi velocity. The blue line and the red line are from different observed times that are 0.1 T and 0.2 T. Considering the real part of the electron’s wave function shown in Fig. 4a, we find that at the time 0.1 T, the electron wave’s spanning is from 0 to $$9.92\,\, \text{nm}$$ approximately, while the electron wave at time 0.2 T extends to around $$14.31 \,\,\text{nm}$$. That is, the wave is dense at the beginning but expands over time. The wave function generally involves electron’s locations propagating along the SWCNT. Accordingly, this result demonstrates the electron transport from the initial point to the tip as time goes on. At first, the probability to find the electron near the center of the tube (initial point) is high. However, afterward this probability for near the center decays while the probability to find the electron at the other point increases since the electron has already moved from the starting point to the tip. Additionally, we discover that the real part of the wave function as shown in Fig. 4a is almost zero in the left side ($$z < 0 $$), thereby confirming that this wave function is for the electron propagating to the right side only. On the other hand, if we simulate the other wave function of the electron transporting in the left direction, that wave function should be zero in the right side as well. Figure 4b shows the phase’s characteristic of the electron wave function that alters as time passes.

**Figure 5:**
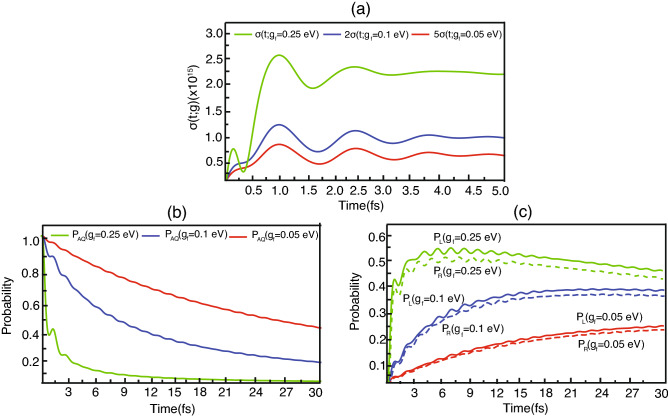
(**a**) The rate of the electron transferred from QD to SWCNT. (**b**) The probability to find the electron at QD. (**c**) The probability to find the electron along SWCNT. Solid lines are for the left side and dot lines are for the right side. The color in every graph: green, blue and red is from the parameter $g_f$ that are 0.25, 0.1 and 0.05 eV, respectively.

In regard to the current density of the probability (of finding the electron along the tube) per Fermi velocity, Fig. 4c portrays the positive current at the right side and negative current at the left side. At the time 0.1 T, the current takes place between about $-9.61 \;  \text{nm}$ and 9.92 nm with the right side’s maximum 5.93 and the left side’s maximum − 6.08, whereas at time 0.2 T, the current emerges from $-13.84$ to 14.31 nm with the right side’s maximum 4.01 and the left side’s maximum $-4.12$, hence affirming the electron transport to both directions over time. Moreover, if we investigate the current density attentively, we find a little asymmetry between the left and right. Considering the arrival of the current density both in green and blue lines, we find out that the current density in the right side expands more than in the left side. On the other hand, the maximum current density of the left side is higher than the right side’s. This reaffirms that the group velocity of electron transport and the probability of finding the electron in each side are different. These characteristics are owing to the asymmetry of energy band structure, thereby making the current density asymmetric because the current density is proportional to the velocity and probability according to Eq. (29). Such the result is in consequence of different group velocity, which is proportional to both special effects as (i) the curvature of the surface of an SWCNT modifies the hopping integral between $\pi$ electrons from the flat graphene and also (ii) curvature-induces $\sigma -\pi$ mixing. More accurately, it has to consider the spin-orbit interaction in the calculation. However, the term can be safely neglected because the energy scale of the factor is sufficiently small^22^. It is consistent with the time-reversal symmetry of the nanotube, which implies that any right-going state has the time-reversed partner, i.e. the left-going state with the same energy and the same absolute value of group velocity. Therefore, if we consider the electron transfer going with equal probabilities from quantum dot to electron states in the *K* and $K'$ valleys of the nanotube, then the total current of both directions is equal. Despite the same total current, this current possesses the two valley degrees of freedom where components from each valley are different as shown in inserted Fig 4c. At time 100 fs, our calculation give the left and right current as 6.08 and 5.93 for *K* valley, respectively. By the time-reversed symmetry, the left and right current should be obtained as 5.93 and 6.08 for $K'$ valley, respectively. Then we obtain the same total current as 12.01 but the left and right current have different composition from each valley degrees of freedom.

Now we consider the rate of the probability of the electron transferring from QD to the SWCNT. According to Eq. (32), such the transfering rate corresponds to the strength of the interaction between QD and SWCNT involving the dipole. That is, more strength of the interaction encourages the transfer of the electron from QD to SWCNT, hence increasing the rate. Figure 5a is the simulation of the transferring rates with the different strength of the interaction entailing to the parameter $g_f$. This simulation demonstrates that the rate of the green line (that is from the highest strength parameter in the samples) is the maximum rate, whereas the rate of the red line (that is from the lowest strength parameter) is the minimum rate. This result illustrates obviously that the more the strength parameter, the more the transferring rate, harmonizing with the theory.

Next we will consider the probabilities of finding the electron in different locations that are QD (Fig. 5b), the left and the right side of the tube (Fig 5c). Considering the probabilities of finding the electron at the QD (green, blue and red lines being from the strength parameter of the interaction $g_f$ that are 0.25, 0.1 and 0.05 eV, respectively), the result portrays that the green line (the highest strength parameter) decay fastest while the red line (the lowest strength parameter) decrease slowest. Then, considering the probability of finding the electron at the tube in both the left and right sides (green, blue and red lines for the strength parameter of the interaction $g_f$ that are 0.25, 0.1 and 0.05 eV, respectively), the green line (the highest strength parameter) grows most rapidly whereas the red line (the lowest strength parameter) grows most slowly. These results demonstrate that when the electron leaves from QD faster, the probability of finding the electron at the tube grows more swiftly. The simulations also display that more strength of the interaction shortens the lifetime of the electron being in QD. Furthermore, comparing the solid and dot lines in Fig. 5c that are the probability of finding the electron in the left and right side of the tube respectively, the probability from the solid lines is more than the dot lines on a whole. This result exhibits that the probability of finding the electron in the left side is more than in the right side. These satisfy the continuity equation Eq. (32) which is composed of the probability source stemming from QD as defined in Eq. (33). Such a tendency is also consistent to the whole Fig. 2 where the group velocity of electrons in the right side is more than in the left side. This different group velocity leads to the different probability in that the probability in the left side is more than in the right side.

While the electron can transfer in two cases (that are QD to SWCNT and SWCNT to QD), our simulation both Fig. 5a,b emphasize the electron transfer from QD to SWCNT case only. Normally, the electron transfer from the QD to the tube is a result of the electron in excited state interacting with the tube, whereas the backward transfer from the tube to the QD is caused by the electron in ground state interacting with the tube. Since the electron in the excited state is generally in a higher orbital compared to the electron in the ground state, and since the electron in the higher orbital has the dipole being stronger than the lower orbital electron, the electron transfer in the excited state has coupling interaction stronger than the electron transfer in the ground state $(g_{f}>> g_{b})$. Therefore, the transfer from QD to SWCNT is much faster than the opposite transfer as the research^36^ shows that the lifetime in the former case is around 10 fs while the latter case is $10\, \text{ps}$ approximately.

## Conclusion

For SWCNT, the electron transferring from QD can transport both in the left and right direction. Nonetheless, the probability (of finding the electron) and group velocity of each direction are different. The simulation in this work focuses on valley-dependent current generation that can be experimentally observed. Our result consequently demonstrates the effects from just one certain valley (*K*) that is impacted by asymmetric band structure. Nevertheless, to derive the effects from the other valley ($K^\prime$), it can be calculated by the time-reversal symmetry. The total current in the left and right hands of SWCNT should, in theory, be equal in absolute value when we take into account the contributions from the *K* and $K'$ valleys. However, the valley degrees of freedom for the right and left currents are composed such that *K* and $K'$ are not identical. Therefore, the left and the right -hand current should store binary information or “valleytronic”. This phenomenon is ascribed by the asymmetric band structure from the curvature effect of specific chiral indexes. The curvature of the surface on an SWCNT modifies the hopping integral between the electrons from the flat graphene and curvature-induces $\sigma -\pi$ mixing on the curved surface of the nanotubes, which is not equal since the differential direction of the electron’s propagation. Therefore, there is an asymmetry of electron transport in SWCNT for certain chiral indexes. The different chiral indexes, moreover, determine the characteristic of the probability and group velocity. Our simulation shows that such asymmetric transport takes place in armchair and chiral types but not in zigzag type. Since the band structure is symmetrical in zigzag type only, the electron transport in this type is symmetrical accordingly. In addition, the electron transport is affected by the spin-orbit interaction. However, this interaction can be neglected since the energy scale of this effect is sufficiently small.

Owing to the difference of the probability (of finding the electron) and group velocity in each direction from particular chiral indexes, the asymmetric current density of the probability generally occurs. Concerning the location of the electron, the probability of finding the electron near the center of the tube is high at initial but it decreases afterwards because the electron has been transported to the tip of the tube over time, hence expanding the wave front of the electron wave function. Additionally, the strength of the interaction from the dipole determines the lifetime of the electron being in QD, thereby encouraging the electron transfer to the tube. The electron in the tube can also transfer reversely to the QD. However, this reverse transfer originates from the interaction of the ground state that is weaker compared to the interaction of the opposite transfer stemming from the excited state since the electron in the excited state is in a higher orbital possessing stronger dipole compared to ground state’s being in lower orbital. Accordingly, the time duration of the electron transferring from the tube to QD is much less compared to the time duration of the opposite transfer. From the investigation of electron transport on SWCNT in our work, it can be applied to carbon nanotube products for various benefits. For example, to adjust artificial antennas created by QD and SWCNT for enhanced light harvesting, we can improve the electrical signal through selecting the chiral index of SWNCT that is suitable for each needed function. Moreover, based on effects from the chiral index demonstrated in this research, our work might be applied for advancing the efficiency of the circuit on solar cells, quantum computers, supercapacitors, transistors and other technologies, especially nanoscale devices.

## Methods

### Asymmetric velocities in SWCNT

The honeycomb lattice can be described in term of two triangular sublattices A and B. The unit vector of the underlying triangular sublattice are chosen to be $\vec {a}_{1}=\frac{a}{2}(\sqrt{3},1)$ and $\vec {a}_{2}=\frac{a}{2}(\sqrt{3},-1)$ with $a=2.46\, A^{0}$ is the lattice constant. Any *A* atom is connected to its nearest-neighbors on *B* sites by the three vectors $\vec {\delta }_{i}$: $\vec {\delta }_{1}= \frac{1}{3}(\vec {a}_{1}+\vec {a}_{2})$ and $\vec {\delta }_{2}= \frac{1}{3}(2\vec {a}_{1}-\vec {a}_{2})$ and $\vec {\delta }_{3}= \frac{1}{3}(-\vec {a}_{1}+\vec {a}_{2})$. For SWCNT, the electronic structure can be discussed by starting point for understating the tight-binding Hamiltonian of a general bipartite lattice with $N_c$ lattice sites $\vec {R}$ and nearest-neighbor lattice vector $\vec {\delta }_{i}$. In this way, geometry of SWCNT is completely specified by a pair of integers (*n*, *m*) denoting the relative position of pair of atoms on graphene strip, $\vec {c}_{n,m}=n\vec {a}_{1}+m\vec {a}_{2}$. When rolled onto each other, form a tube. This chiral vector $\vec {c}_{n,m}$ defines the circumference of the tube axis. The radius $\rho (n,m)$ and the chiral angle $\theta (n,m)$ of the SWCNT can thus be estimated from

1 $$\begin{aligned} \rho (n,m)=\frac{a}{2\pi }\sqrt{n^2+nm+m^2} \end{aligned}$$

and

2 $$\begin{aligned} \theta (n,m)=arccos\left[ \frac{2n+m}{2\sqrt{n^2+nm+m^2}}\right] \end{aligned}$$

the chiral angle $\theta (n,m)$ is in the range $0\le \theta (n,m) \le 30^\circ$, because of the hexagonal symmetry of the graphene lattice. This chiral angle also denotes the tilt angle of the hexagons for the direction of the nanotube axis. Zigzag tubes of the type $\theta (n,0)=0^\circ$ exhibit a zigzag pattern along the circumference. For metallic SWCNT, an armchair tube of the type $\theta (n,n)=30^\circ$ exhibits an armchair pattern along the circumference. Both zigzag and armchair nanotube is a chiral tube, in contrast to a general chiral tube where $m \ne n \ne 0$.

For the tilted dispersion relation of SWCNT, it is discussed by a curvature effect tight-binding Hamiltonian of an electron in terms of a two-by-two matrix in the $\vec {k}$ space:

3 $$\begin{aligned} {{\textbf {H}}}_{SC}= \sum _{\vec {k}}\left( \begin{array}{cc} {\hat{c}}^{\dagger }_{c,\vec {k}} &{} {\hat{c}}^{\dagger }_{v,\vec {k}} \\ \end{array} \right) {\mathscr {H}}_{\text {0}}\left( \begin{array}{c} {\hat{c}}_{c,\vec {k}} \\ {\hat{c}}_{v,\vec {k}} \\ \end{array} \right) , \end{aligned}$$

where *c* and *v* are the band index for conduction and valence band, respectively. The creation ${\hat{c}}^{\dagger }_{c(v)\vec {k}}$ and annihilation ${\hat{c}}_{c(v),\vec {k}}$ operator of the electron in conduction(valence) band of SWCNT with the momentum $\hbar \vec {k}$, respectively. The single-particle Hamiltonian describes a curvature effect of the nanotube. Two characteristic effects are discussed to be i) the curvature of the surface of an SWNT modifies the hopping integral between $\pi$ electrons from the flat graphene and also ii) curvature-induces $\sigma -\pi$ mixing. For curvature-induced $\sigma -\pi$ mixing together with Slater-Koster type projection for $\pi -\pi$ and $\sigma -\pi$ hopping integrals on the curved surface of the nanotubes, correction terms up to the first order of *k* are derived by using a second-order perturbation technique. Following curvature-induced tilting of the linear bands Hamiltonian^22,23^, which gives the asymmetric velocities, the Hamiltonian ${\mathscr {H}}_{\text {0}}(\vec {k})$ is given as

4 $$\begin{aligned} {\mathscr {H}}_{\text {0}}= \sum _{\mu =0,1,2} \hbar \vec {v}_{\mu }{\varvec{{\sigma }}}^{\mu }\cdot \vec {k}+A^{(pse)}_{\mu }\sigma ^{\mu }, \end{aligned}$$

and $A^{(pse)}_{\mu }\sigma ^{\mu }= v_{F}(\tau \Delta _{1}{\varvec{{\sigma }}}_{1}+\Delta _{2}{\varvec{{\sigma }}}_{2})$ is the pseudo vector potential and $\vec {k}=(k_{\phi },k_{z})$ and ${{\textbf {I}}}$ is the identity matrix and ${\varvec{{\sigma }}}_{1(2)}$ are the Pauli matrices,

5 $$\begin{aligned} {\varvec{{\sigma }}}_{1}=\left( \begin{array}{cc} 0 &{} 1 \\ 1 &{} 0 \\ \end{array} \right) , {\varvec{{\sigma }}}_{2}=\left( \begin{array}{cc} 0 &{} -i \\ i &{} 0 \\ \end{array} \right) . \end{aligned}$$

Here the velocities $\vec {v}_{\mu }=(v_{\phi ,\mu },v_{z,\mu })$ are defined as a function of parameters as $\varepsilon _{2s}$ the energy of the 2*s* orbital with $V_{sp}$, $V^{\sigma }_{pp}$ and $V^{\pi }_{pp}$ hopping transfer integrals between two neighboring for the 2*s*, $\sigma$ and $\pi$ orbitals in flat two dimensional graphence, respectively. The shift of wave number by the curvature effect, $\Delta _{1}$ and $\Delta _{2}$ are given by

6 $$\begin{aligned} \Delta _{1}=4\beta cos(3\theta )/\rho ^2 \,\,,\,\,\Delta _{2}=4\xi sin(3\theta )/\rho ^2 \end{aligned}$$

where the coefficients $\beta =0.00436$ nm and $\xi =-0.0185$ nm are given analytically in a simpler tight-binding model from Izumida^22,23^. For experimental aspects concerning spin-orbit coupling in CNT, it was studied as^37^.

Using gauge transformation, the general solution of the single-particle Hamiltonian, Eq. (6) yields the energy dispersion

7 $$\begin{aligned} E^{(\ell )}_{\kappa }(\vec {k})= \sum _{i=\phi ,z}\tau \hbar v_{i,0}k_{i} +\kappa \Vert \sum _{i=\phi ,z}\hbar (v_{i,1}-i\tau v_{i,2})k_{i} \Vert \end{aligned}$$

where $\kappa$ plays the role of the band index for $\kappa = 1$ conduction or $\kappa = -1$ valence band. The index $\tau$ denotes the $K(\tau = 1)$ or $K'(\tau = -1)$ valleys, hence making the different sign of the wave function at lattice points designated by + or − signs, see in Eq. (8). In addition, a boundary condition is imposed that yield the appropriate quantization of the vector $\vec {k}$. The SWCNT is obtained by rolling a graphene layer into a tube, in the angular direction, its wave function always obeys periodic boundary condition $\Psi (\vec {C}_{nm})=exp(i\vec {k}\cdot \vec {C}_{nm})\Psi (0)=e^{i2{\pi }\ell }\Psi (0)$ . This boundary condition leads to a quantization of the transverse component of the vector $\vec {k},\,\, k_{\phi }=2\pi \ell /\mid \vec {C}_{nm}\mid$, with $\ell$ being an integer. Analogous to the spinor, the two components of eigenstates can be written as

8 $$\begin{aligned} |\chi ^{(\ell )}_{\kappa }(\vec {k})\rangle = \frac{1}{\sqrt{2}} \left( \begin{array}{c} \kappa e^{i \Phi _{\kappa }(\vec {k})}\\ 1 \\ \end{array} \right) . \end{aligned}$$

The phase factor of spinor $\Phi (\vec {k_{\kappa }})$, depending on the Weyl-Dirac velocities as Eqs. (6)–(9) is given as

9 $$\begin{aligned} \Phi (\vec {k})= Arg\left( (v_{\phi ,1}-i\tau v_{\phi ,2})k_{\phi }+(v_{z,1}-i\tau v_{z,2})k_{z} \right) . \end{aligned}$$

The asymmetric velocities is obtained by differentiating Eq. (9) with respect to $k_{z}$ as

10 $$\begin{aligned} v^{(\kappa )}_{\ell ,L}=-\frac{1}{\hbar }\frac{\partial }{\partial q}E^{\kappa }(\vec {k})\,\, (q<0), \,\,\,\,\,\, v^{(\kappa )}_{\ell , R}=\frac{1}{\hbar }\frac{\partial }{\partial q}E^{\kappa }(\vec {k}) \,\,(q>0). \end{aligned}$$

where $q=k_{z}-k_{0z}$ is a relative wave number with a minimum point $k_{0z}$ in the band structure. The asymmetric velocities can be approximated as (see the supplement)

11 $$\begin{aligned} v^{(K)}_{L}=\sqrt{v^{2}_{z,1}+v^{2}_{z,2}}- \tau \Delta v,\,\, v^{(K)}_{R}=\sqrt{v^{2}_{z,1}+v^{2}_{z,2}}+\tau \Delta v \end{aligned}$$

where the different of $\Delta v$ is given as

12 $$\begin{aligned} \Delta v= \frac{a^3 \varepsilon _{2s}V^{\pi }_{pp}( V^{\sigma }_{pp}-V^{\pi }_{pp})}{6\sqrt{3} \hbar \rho ^2 V^{2}_{sp}}sin(3\theta ). \end{aligned}$$

Additionally, to derive the specific variables of the other valley ($K^\prime$), it can be theoretically obtained by the time-reversal symmetry.

**Figure 6:**
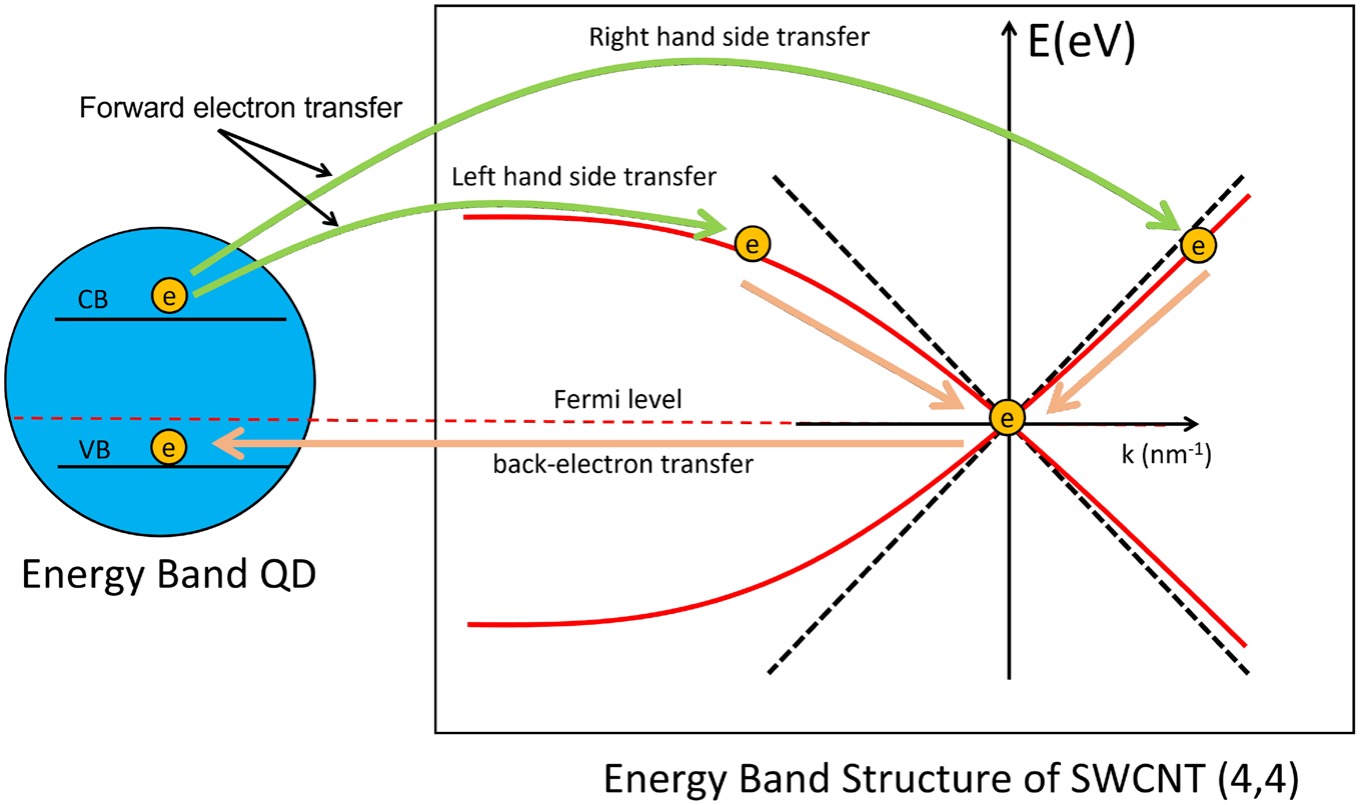
Schematized models of forward- and backward-electron transfer between QD and SWCNT (4,4). The dot line is an symmetrical energy band structure for zigzag type (4,0). For forward-electron transfer from QD to SWCNT, there are two possible ways of electron (Green lines) that are (i) right hand side and (ii) left hand side transfer. The pink line is backward electron transfer from SWCNT to QD.

### Probability for finding the transferred charge from QD to SWCNT

In this section, we investigate a photoinduced current event that quantum dot absorb photon creating electron-hole pair, represented by an exciton state and ground state showed in schematic illustration of Fig. 6. Let us consider an electron transition process following that (i), QD could be activated by visible light to generate electrons and holes, and (ii) the holes are scavenged by ethanol and the electron completely transferring to the conduction band of the *K* valleys, leading to an enhanced photocurrent intensity due to the electronic band structure of SWCNT. Finally, (iii) the back-electron transfer from SWCNT to QD. Furthermore, by the time-reversal symmetry, the calculation from the other valley transition ($K^\prime$) can be derived. Nonetheless, to investigate the valley-dependent current, we emphasize the result from one specific valley.

Following the mathematical discussion in ref.^28,29^, the composite system is considered as the transition process of electron to SWCNT interaction, having defined the system Hamiltonian as

13 $$\begin{aligned} {{\textbf {H}}}= {{\textbf {H}}}_{SC}+{{\textbf {H}}}_{QD}+{{\textbf {H}}}_{SC-QD}. \end{aligned}$$

With an understanding of quantum confinement^38^, the exciton is described by the Hamiltonian ${{\textbf {H}}}_{QD}$ with the excited state $|ex\rangle$ and $|g\rangle$ the ground state,

14 $$\begin{aligned} {{\textbf {H}}}_{QD}= \hbar (\omega _{ex}-i\Gamma /2) {\hat{a}}^{\dag }_{ex}{\hat{a}}_{ex} +\hbar \omega _{g} {\hat{a}}^{\dag }_{g}{\hat{a}}_{g}. \end{aligned}$$

where $\Gamma$ is the lifetime of exciton state and the electron creation ${\hat{a}}^{\dag }_{ex} ({\hat{a}}^{\dag }_{g})$ and annihilation ${\hat{a}}_{ex} ({\hat{a}}_{g})$ operators in the excited state $|ex\rangle$ ($|g\rangle$ the ground state) for the QD system. Schematized models of forward- and backward-electron transfer from CDs to SWCNTs with the related characteristic timescales as obtained by a fitting procedure^36^ . Femtosecond transient absorption confirms indeed an ultrafast $(< 100 \; \text{fs})$ electron transfer independent of nanotubes being conductive or semiconductive in nature, followed by a much slower back electron transfer $(\approx 60 \; \text{ps})$ from the nanotube to QD. Following the dipole-dipole interaction between a quantum dot and surface plasmons nanowires and a graphene nanodisk^28,38,39^, the charge-transfer interaction between a quantum dot nano particle and SWCNT is given in the form

15 $$\begin{aligned} {{\textbf {H}}}_{SC-QD}= \int dz \delta (z)\left[ g_{f}({\hat{R}}^{\dag }(z)+{\hat{L}}^{\dag }(z)){\hat{a}}_{ex} + g_{b}( {\hat{R}}(z)+{\hat{L}}(z)){\hat{a}}^{\dag }_{g} \right] \end{aligned}$$

where $g_{f}$ and $g_{b}$ are a coupling parameter between QD and SWCNT which discuss forward- and back-electron transfer from CD to SWCNT, respectively. These coupling parameters correspond with dipole interaction between electron states in QD and the field of the tube. Therefore, the coupling parameters depend on QD shape and the distance between the dot and the nanotube. These effects were studied in^28,40–42^. The dipole coupling $g_f$ of right- and left-going states may not be the same since each state has generally different wave functions at the same |*q*| (or at the same energies). If we determine the different dipole coupling parameters, the current will definitely be asymmetric. Nevertheless, since we investigate the band structure effects intently, we assume that the coupling parameter of both directions is the same as the theoretical works^39,43,44^. We, on this ground, can determine the strength of the interaction between QD and SWCNT in each direction by these two parameters. Based on the work^42^ , the interaction of forward-electron transfer is generally more than the interaction of back-electron transfer since the former has linearly polarized dipoles but the latter has degenerate 2D planar dipoles whose average is zero, thereby $g_{f}>> g_{b}$ normally. In addition, the right- and left-going electron field operators are defined respectively in the form as^28,29^

16 $$\begin{aligned} {\hat{R}}(z)= \sqrt{\frac{L}{2\pi } }\int ^{\infty }_{0} dq\,\,e^{-iqz}{\hat{c}}_{c,q} |\chi _{c}(q)\rangle \,\,\, \text {and} \,\,\,{\hat{L}}(z)= \sqrt{\frac{L}{2\pi } }\int ^{0}_{-\infty }dq\,\, e^{-iqz}{\hat{c}}_{c,q} |\chi _{c}(q)\rangle . \end{aligned}$$

These are the Dirac spinor field expanded in terms of annihilation operators with spinor valued coefficients. For a plane-wave state normalization, we employ the trick of normalizing them in a large (relative to potential range) SWCNT of side *L*.

Now we consider the probability for finding the electron transferring from QD to the conduction band of SWCNT. To calculate the probability, we solve the time-dependent perturbation by expanding the time-evolution operator. The expansion is as the following scheme:

17 $$\begin{aligned} {{\textbf {U}}}(t,0) =e^{-\frac{i}{\hbar }{{\textbf {H}}}_{SC}t}\sum ^{\infty }_{n=0}\frac{(-i)^{n}}{n!\hbar ^{n}}\int _{0}^{t} dt'\cdots \int _{0}^{t} dt^{(n)}{{\textbf {T}}}[{{\textbf {H}}}_{I}(t')\cdots {{\textbf {H}}}_{I}(t^{(n)})], \end{aligned}$$

where ${{\textbf {H}}}_{I}$ is the interaction Hamiltonian in Dirac picture and ${{\textbf {T}}}$ is the time-ordering operator,

18 $$\begin{aligned} {{\textbf {H}}}_{I}={{\textbf {H}}}_{SC-QD}+{{\textbf {H}}}_{QD}. \end{aligned}$$

Once, we define the measured state which find an electron moving as right-going $(z > 0)$ or left-going $(z < 0)$ in the conduction band with the band index $\ell =0$ (low energy conduction band),

19 $$\begin{aligned} |R(z)\rangle _{0} = {\hat{R}}^{\dag }(z) |0_{ex}, \mu _{SC}\rangle \,\,\, \text {and} \,\,\, |L(z)\rangle _{0} = {\hat{L}}^{\dag }(z)|0_{ex}, \mu _{SC}\rangle \,\,\, \text {and}\,\,\, |QD,1_{ex}\rangle _{0}=|1_{ex}, \mu _{SC}\rangle \,\,\,. \end{aligned}$$

It means that the electron transfers from QD to the conduction band having the index $\ell =0$ and finding the electron in the right and left-hand side of SWCNT. Here $|0_{ex},\mu _{SC}\rangle =|0 _{ex}\rangle \bigotimes |\mu _{SC}\rangle$ and $|1_{ex},\mu _{SC}\rangle =|1_{ex}\rangle \bigotimes |\mu _{SC}\rangle$ are tensor product quantum states between the vacuum state $|0_{ex}\rangle$ and the occupation $|1_{ex}\rangle$ of an electron for the excited state of the quantum dot, and $|\mu _{SC}\rangle$ a quantum state of the electron belong to SWCNT, respectively.

For the convenience of calculation, the back-electron transfer from SWCNT to CD is neglected. Now, probability amplitude of an event, the electron transferring from QD to the conduction band having the index $\ell$ of SWCNT and finding the electron in the right and left-hand side can be obtained as

20 $$\begin{aligned} A_{R}(z,t) =\, _0\langle R^{(\ell )}(z)| e^{-\frac{i}{\hbar }{{\textbf {H}}}t}|1_{ex}, \mu _{SC}\rangle \,\,\,\,\,\, \text {and}\,\,\,\,\,\, A_{L}(z,t)=\, _0\langle L^{(\ell )}(z)|e^{-\frac{i}{\hbar }{{\textbf {H}}}t}|1_{ex}, \mu _{SC}\rangle ,\,\,\, \text {respectively.} \end{aligned}$$

In addition, a probability amplitude of finding the electron is in the excited state in QD is given as

21 $$\begin{aligned} A_{QD}(0,t) =\, _{0}\langle QD,1_{ex}| e^{-\frac{i}{\hbar }{{\textbf {H}}}t}|QD,1_{ex}\rangle _{0}. \end{aligned}$$

To evaluate a probability amplitude of finding the electron in the left and right-hand side, we employ the assumption that the coupling is weak. So, in each term of the Dyson’s series, we keep only the terms of first-order in $g_f$. Using two following summation formulae^28^, we have

22 $$\begin{aligned} \frac{1}{1-x}=1-x+x^{2}-x^{3}+\cdots, \; \text {and}\,\,\,\, \frac{e^{ab}}{1+a} = 1+a(1-b)+a^{2}(1-b+\frac{b^{2}}{2!})+a^{3}(1-b+\frac{b^{2}}{2!}-\frac{b^{3}}{3!})+\cdots . \end{aligned}$$

Equation (21), probability amplitude of an event, the electron transferring from QD to the conduction band having the index $\ell$ of SWCNT become approximately

23 $$\begin{aligned}  A_{R}(z,t) & = g_{f}\sqrt{L}\int _{0}^{\infty } \frac{dq}{2\pi } \frac{e^{iq z}(e^{-\frac{i}{\hbar }E_{c}(q)t}-e^{-i(\omega _{ex}-\frac{i}{2}\Gamma )t})}{E_{c}(q)-\hbar (\omega _{ex}-\frac{i}{2}\Gamma )} \,\,\,\text {and} \,\,\,\, \\ A_{L}(z,t)\nonumber  & = g_{f}\sqrt{L}\int _{-\infty }^{0} \frac{dq}{2\pi }\frac{e^{i q z}(e^{-\frac{i}{\hbar }E_{c}(q)t}-e^{-i(\omega _{ex}-\frac{i}{2}\Gamma )t})}{E_{c}(q)-\hbar (\omega _{ex}-\frac{i}{2}\Gamma )}. \end{aligned}$$

Probability amplitude of finding the electron in the excited state in QD can be approximated in the form

24 $$\begin{aligned} A_{QD}(t)=e^{i(\omega _{ex}-\frac{i}{2}\Gamma )t}-ig_{f} \sqrt{L}\sum _{\eta =R,L}A_{\eta }(0,t)/\hbar k^{(c)}_{\eta }v_{\eta } \end{aligned}$$

where $k^{(c)}_{\eta }=(\omega _{ex}-\frac{i}{2}\Gamma )/v_{\eta }$ is the complex wave number and $\eta =L (-1),\,\,\,R(+1)$ is the left and right-hand side index, respectively. For considering the armchair nanotubes (*n*, *n*) and the integer $\ell =0$, energy gap is very small comparing in region $|E|< 1$ eV. It is a different slope for linear bands, called linear band tilting. Following the method proposed by Ref.^28,29^, we can further extend the range of $q\in [0,\infty )$ (right- going electron) and $q\in (\infty ,-0)$ (left-going electron). With energy dispersion Eq. (9), thus, the curvature effect Hamiltonian in Eq. (3) can be rewritten in the form of creation (annihilation) right- and left-going electron at *z*,

25 $$\begin{aligned} A_{R}(z,t) &  =\left( \frac{g_{f}\sqrt{L}}{\hbar v_{R}}\right) \int _{0}^{\infty } \frac{dq}{2\pi } \frac{e^{i q (z-v_{R} t)}-e^{i(q z-(\omega _{ex}-\frac{i}{2}\Gamma )t)}}{q-(\omega _{ex}-\frac{i}{2}\Gamma )/v_{R}} \,\,\,\text {and} \,\,\,\,\\ A_{L}(z,t)\nonumber  & =\left( \frac{g_{f}\sqrt{L}}{\hbar v_{L}}\right) \int _{-\infty }^{0} \frac{dq}{2\pi } \frac{e^{i q (z-v_{L} t)}-e^{i(q z-(\omega _{ex}-\frac{i}{2}\Gamma )t)}}{q-(\omega _{ex}-\frac{i}{2}\Gamma )/v_{L}}. \end{aligned}$$

Here it is to be noted that $\Gamma (s,x)= \int _{x}^{\infty } dq \,\,\, q^{s-1}e^{-q}$ is the upper incomplete gamma function. Next, we can rewrite the electron in the left and right-hand side probability amplitude, Eq. (25) as

26 $$\begin{aligned} A_{\eta }(z,t)=\left( \frac{g_{f}\sqrt{L}}{2\pi \hbar v_{\eta }} \right) e^{i k^{(c)}_{\eta }(\eta z-v_{\eta } t)}\left( \Gamma (0,ik^{(c)}_{\eta }(\eta z -v_{\eta } t))- \Gamma (0,i\eta z k^{(c)}_{\eta })\right) , \end{aligned}$$

Now we can discuss the dynamical of electron by separating into three composition as the right or left-going state and occupation in the excited state in QD,

27 $$\begin{aligned} |\Psi (t)\rangle = \int dz \left[ A_{R}(z,t) |R(z)\rangle _{0} +A_{L}(z,t) |L(z)\rangle _{0}\right] + A_{QD}(t)|QD,1_{ex}\rangle _{0}. \end{aligned}$$

It implies that the transferred electron from QD into two distinct components as the right and left-hand side electron state in SWCNT, a phenomenon called space quantization. The probability of finding the electron on the left and right-hand side as Fig. 2 is an absolute square of $A_{L}(z,t)$ and $A_{R}(z,t)$, respectively. Now, we focus on the currents and transfer probability between the quantum dot and SWCNT. It has been pointed out that the conventional current density usually following^45,46^ and can be derived as this operator from the standard quantum-mechanical continuity equation modified by taking into account Dirac-like equation for electrons in the nanotube, (see the supplement) defined as

28 $$\begin{aligned} J^{(K)}_{\eta }(z,t) = \,_{\eta }\langle \Phi (z,t)| \hat{{{\textbf {v}}}}_{z}|\Phi (z,t)\rangle _{\eta }\,\,\,\,\,\,\text {with}\,\,\,\, |\Phi (z,t)\rangle _{\eta }=A_{\eta }(z,t) |\eta (z)\rangle _{0}\,\,\,\,\, \text {and} \,\,\,\,\, {{\textbf {v}}}_{z}=\frac{i}{\hbar } [{{\textbf {H}}},z]. \end{aligned}$$

To calculate current density, we use the relation

29 $$\begin{aligned} v_{R}=\,_{0}\langle R(z)|\hat{{{\textbf {v}}}}_{z}|R(z)\rangle _{0} \,\,\, \text {and}\,\,\,\,v_{L}=-\,_{0}\langle L(z)|\hat{{{\textbf {v}}}}_{z}|L(z) \rangle _{0} \end{aligned}$$

and insert Eq. (29) to Eq. (28). Then we obtain current density in the form of probability times velocity

30 $$\begin{aligned} J^{(K)}_{\eta }= \eta v_{\eta }P_{\eta }(z,t) \,\,\,\,\,\,\text {with}\,\,\,\, \eta =1(R) \,\,\,\,\,\,\text {or}\,\,\,\,-1(L). \end{aligned}$$

Here $P_{\eta }(z,t)$ is the probability density (probability per length) of finding the electron on the left and right-hand side, and $P_{QD}(0,t)$ is the probability of finding the electron in QD,

31 $$\begin{aligned} P_{\eta }(z,t)= |A_{\eta }(z,t)|^{2} \,\,\,\, \text {and}, \,\, P_{QD}(t)= |A_{QD}(t)|^{2}. \end{aligned}$$

To calculate electron transport and transfer between the quantum dot and SWCNT, we chose the modified normalization condition as

32 $$\begin{aligned} \int ^{L/2}_{-L/2} dz \left( P_{R}(z,t)+P_{L}(z,t) \right) + P_{QD}(t)= e^{-\Gamma t}, 
\end{aligned}$$

corresponding with non-Hermitian Hamiltonian Eq. (15). Thus, by considering the change transfer between QD and SWCNT, the continuity equation for the probability density is written as the divergence theorem to obtain

33 $$\begin{aligned} \sum _{\eta =L,R}\left( \frac{\partial }{\partial z} J^{(K)}_{\eta }(z,t) +\frac{\partial }{\partial t} P_{\eta }(z,t)\right) =\sigma (t) \delta (z), \end{aligned}$$

where $\sigma (t)$ is the generation of the probability for electron transferred form QD to SWCNT per unit time, referred to as a “sources.” being in the form that

34 $$\begin{aligned} \sigma (t)= \frac{g_{f}}{\hbar }\sum _{\eta =L,R} 2\,Im\left[ A^{*}_{\eta }(0,t)A_{QD}(t)\right] +\Gamma P_{QD}(t)+ \frac{\partial }{\partial t}P_{QD}(t). \end{aligned}$$

## Supplementary Information

Supplementary Information.

## Data Availability

The data sets used and analyzed during the current study available from the corresponding author (S. Boonchui) on reasonable request.
