# Supplementary material for: Investigating valley-dependent current generation due to asymmetric energy dispersion for charge-transfer from a quantum dot to single-walled carbon nanotube
Source: Sci Rep. 2023 Feb 22;13:3105. doi: 10.1038/s41598-023-30247-1 (PMC9947177; doi:10.1038/s41598-023-30247-1)
Supplement: Supplementary file 1 — Supplementary Information. [file 41598_2023_30247_MOESM1_ESM.pdf]

Supplemental document

1. ASYMMETRIC VELOCITIES IN SWCNT

For the tilted dispersion relation of SWCNT, It is discussed by a curvature effect tight-binding Hamiltonian of an electron in terms of a two-by-two matrix in the $\vec{k}$ space:

$$\mathbf{H}_{SC} = \sum_{\vec{k}} \begin{pmatrix} \hat{c}^{\dagger}_{c,\vec{k}} & \hat{c}^{\dagger}_{v,\vec{k}} \end{pmatrix} \mathcal{H}_0 \begin{pmatrix} \hat{c}_{c,\vec{k}} \\ \hat{c}_{v,\vec{k}} \end{pmatrix}, \tag{S1}$$

where c and v are the band index for conduction and valence band, respectively. The creation $\hat{c}^{\dagger}_{c(v)\vec{k}}$ and annihilation $\hat{c}_{c(v),\vec{k}}$ operator of the electron in conduction(valence) band of SWCNT with the momentum $\hbar\vec{k}$, respectively. The single-particle Hamiltonian describes a curvature effect of the nanotube gives the asymmetric velocities, the Hamiltonian $\mathcal{H}_0(\vec{k})$ is given as

$$\mathcal{H}_0 = \sum_{\mu=0,1,2} \hbar\vec{v}_{\mu}\sigma^{\mu} \cdot \vec{k} + A^{(pse)}_{\mu}\sigma^{\mu}, \tag{S2}$$

and $A^{(pse)}_{\mu}\sigma^{\mu} = v_F(\tau\Delta_1\sigma_1 + \Delta_2\sigma_2)$ is the pseudo vector potential and $\vec{k} = (k_{\phi}, k_z)$ and $\mathbf{I}$ is the identity matrix and $\sigma_{1(2)}$ are the Pauli matrices,

$$\sigma_1 = \begin{pmatrix} 0 & 1 \\ 1 & 0 \end{pmatrix}, \sigma_2 = \begin{pmatrix} 0 & -i \\ i & 0 \end{pmatrix}. \tag{S3}$$

Here the velocities $\vec{v}_{\mu} = (v_{\phi,\mu}, v_{z,\mu})$ are defined as a function of parameters as ϵ_{2s} the energy of the $2s$ orbital with V_{sp}, V^{σ}_{pp} and V^{π}_{pp} hopping transfer integrals between two neighboring for the $2s$, σ and π orbitals in flat two dimensional graphence, respectively.

$$\vec{v}_0 = \tau(v_{\phi,0}, v_{z,0}) = 2\tau(\frac{c_5}{\rho^2}cos3\theta, \frac{c_6}{\rho^2}sin3\theta) \tag{S4}$$

$$\vec{v}_1 = (v_{\phi,1}, v_{z,1}) = (v_F + \frac{2}{\rho^2}(c_1 + c_3cos6\theta), 2\frac{c_3}{\rho^2}sin6\theta) \tag{S5}$$

$$\vec{v}_2 = \tau(v_{\phi,2}, v_{z,2}) = \tau(2\frac{c_5}{\rho^2}cos3\theta, v_F + \frac{2}{\rho^2}(c_2 - c_4cos6\theta)) \tag{S6}$$

Following ref. [22,23], the coefficients in Eq.(S4)-Eq.(S6) are given as

$$c_1 = \frac{a^3}{16\sqrt{3}\hbar}\left[\frac{5(V^{\pi}_{pp})^2 - 6V^{\pi}_{pp}V^{\sigma}_{pp} + 5(V^{\sigma}_{pp})^2}{V^{\pi}_{pp} + V^{\sigma}_{pp}}\right] \tag{S7}$$

$$c_2 = \frac{a^3}{16\sqrt{3}\hbar}\left[\frac{-7(V^{\pi}_{pp})^2 + 10V^{\pi}_{pp}V^{\sigma}_{pp} + (V^{\sigma}_{pp})^2}{V^{\pi}_{pp} + V^{\sigma}_{pp}}\right] \tag{S8}$$

$$c_3 = -c_4 = \frac{a^3}{32\sqrt{3}\hbar}(7V^{\pi}_{pp} - V^{\sigma}_{pp}) \tag{S9}$$

$$c_5 = -c_6 = \frac{a^3}{6\sqrt{3}\hbar}(\frac{\epsilon_{2s}V^{\pi}_{pp}(V^{\pi}_{pp} - V^{\sigma}_{pp})}{(V_{sp})^2}) \tag{S10}$$

The shift of wave number by the curvature effect, Δ_1 and Δ_2 are given by

$$\Delta_1 = 4\beta cos(3\theta)/\rho^2 \ , \ \Delta_2 = 4\xi sin(3\theta)/\rho^2 \tag{S11}$$

where the coefficients $\beta = 0.00436$ nm and $\xi = -0.0185$ nm are given analytically in a simpler tight-binding model. Using gauge transformation, the general solution of the single Hamiltonian, Eq.(S2) yields the energy dispersion

$$E_\kappa^{(\ell)}(\vec{k}) = \sum_{i=\phi,z} \tau\hbar v_{i,0} k_i + \kappa \| \sum_{i=\phi,z} \hbar(v_{i,1} - i v_{i,2}) k_i \| \tag{S12}$$

where κ plays the role of the band index for $\kappa = 1$ conduction or $\kappa = -1$ valence band. The index τ denotes the $K(\tau = 1)$ or $K'(\tau = -1)$ valleys, hence making the different sign of the wave function at lattice points is designated by + or - signs, see in Eq. (S13). In addition, a boundary condition is imposed that yield the appropriate quantization of the vector $\vec{k}$. The SWCNT is obtained by rolling a graphene layer into a tube, in the angular direction its wave function always obey periodic boundary condition $\Psi(\vec{C}_{nm}) = exp(i\vec{k} \cdot \vec{C}_{nm})\Psi(0) = e^{i2\pi\ell}\Psi(0)$.

This boundary condition leads to a quantization of the transverse component of the vector $\vec{k}$, $k_\phi = 2\pi\ell / \mid \vec{C}_{nm} \mid$, with ℓ being an integer. Analogous to the spinor, the two components eigenstates can be written as

$$|\chi_\kappa^{(\ell)}(\vec{k})\rangle = \frac{1}{\sqrt{2}} \begin{pmatrix} \kappa e^{i\Phi_\kappa(\vec{k})} \\ 1 \end{pmatrix}. \tag{S13}$$

The phase factor of spinor $\Phi(\vec{k_\kappa})$, depending on the Weyl-Dirac velocities as Eqs. (6)-(9) is given as

$$\Phi(\vec{k}) = Arg\left((v_{\phi,1} - i\tau v_{\phi,2})k_\phi + (v_{z,1} - i\tau v_{z,2})k_z\right). \tag{S14}$$

The asymmetric velocities is obtained by differentiating Eq. (S14) with respect to k_z as

$$v_{\ell,L}^{(K)} = -\frac{1}{\hbar}\frac{\partial}{\partial q}E^\kappa(\vec{k}) \ (q < 0), \quad v_{\ell,R}^{(K)} = \frac{1}{\hbar}\frac{\partial}{\partial q}E^\kappa(\vec{k}) \ (q > 0). \tag{S15}$$

where $q = k_z - k_{0z}$ is a relative wave number with a minimum point k_{0z} in the band structure. Inserting Eq.(S12) for $\kappa = 1$ into Eq.(S15) and using the linear band approximation, we have

$$v_{\ell,L}^{(K)} = -\frac{1}{\hbar}\frac{\partial}{\partial q}\left(\tau\hbar v_{z,0}k_z - \|\hbar(v_{z,1} - iv_{z,2})\|k_z\right) = -\tau v_{z,0} + \|(v_{z,1} - iv_{z,2})\| \ ; (q < 0), \tag{S16}$$

and

$$v_{\ell,R}^{(K)} = \frac{1}{\hbar}\frac{\partial}{\partial q}\left(\tau\hbar v_{z,0}k_z + \|\hbar(v_{z,1} - iv_{z,2})\|k_z\right) = \tau v_{z,0} + \|(v_{z,1} - iv_{z,2})\| \ ; (q > 0). \tag{S17}$$

So, the asymmetric velocities can be approximated as

$$v_L^{(K)} = \sqrt{v_{z,1}^2 + v_{z,2}^2} - \tau\Delta v, \ v_R^{(K)} = \sqrt{v_{z,1}^2 + v_{z,2}^2} + \tau\Delta v \tag{S18}$$

where the different of Δv is given as

$$\Delta v = v_{z,0} = \frac{a^3\epsilon_{2s}V_{pp}^\pi(V_{pp}^\sigma - V_{pp}^\pi)}{6\sqrt{3}\hbar\rho^2 V_{sp}^2} sin(3\theta). \tag{S19}$$

Additionally, to derive the specific variables of the other valley (K'), it can be theoretically obtained by the time-reversal symmetry.

2. THE CURRENT PROBABILITY DENSITY

Now we will discuss the dynamics of electrons by separating into three composition as the right or left-going state and occupation in the excited state of QD,

$$|\Psi(t)\rangle = \int dz\left[A_R(z,t)|R(z)\rangle_0 + A_L(z,t)|L(z)\rangle_0\right] + A_{QD}(t)|QD, 1_{ex}\rangle_0. \tag{S20}$$

This implies that the electron transfers from QD into two distinct components as the right and left-hand side electron state in SWCNT, hence leading to a phenomenon called space quantization.

The probability of finding the electron on the left and right-hand side as Fig. (2) is an absolute square of $A_L(z,t)$ and $A_R(z,t)$, respectively.

Next, we focus on currents and change transfer between the QD and SWCNT. It has been pointed out that the conventional current density usually defined as

$$J_\eta^{(K)}(z,t) = {}_\eta\langle\Phi(z,t)|\hat{\mathbf{v}}_z|\Phi(z,t)\rangle_\eta \quad \text{with} \quad |\Phi(z,t)\rangle_\eta = A_\eta(z,t)|\eta(z)\rangle_0 \quad \text{and} \quad \mathbf{v}_z = \frac{i}{\hbar}[\mathbf{H},z]. \tag{S21}$$

Then, we have defined the charge current in such a way that the dimension of $J_\eta(z,t)$ is that of charge times velocity. Since the motion is constrained to the nanotube, only two spatial components of $\hat{\vec{\mathbf{v}}}$ are needed, $\hat{\mathbf{v}}_\phi = \frac{i\rho}{\hbar}[\mathbf{H}_{SC},\phi]$ and $\hat{\mathbf{v}}_z = \frac{i}{\hbar}[\mathbf{H}_{SC},z]$. Inserting Eq. (18) and Eq. (27) to Eq.(29), we obtain the current density as

$$J_\eta(z,t) = \eta v_\eta P_\eta(z,t), \tag{S22}$$

where $P_\eta(z,t)$ and $P_{QD}(0,t)$ is the probability density of finding the electron on the left and right-hand side, and the probability of finding the electron in QD, respectively

$$P_\eta(z,t) = |A_\eta(z,t)|^2 \ \text{ and, } \ P_{QD}(0,t) = |A_{QD}(0,t)|^2. \tag{S23}$$

These are the Dirac spinor field expanded in terms of annihilation operators with spinor valued coefficients. Accordingly, the probability amplitude of an event, the electron transferring from QD to the conduction band having the index ℓ of SWCNT and finding the electron in the right and left-hand side, can be obtained respectively as

$$A_R(\vec{r},t) = {}_0\langle R^{(\ell)}(z)|e^{-\frac{i}{\hbar}\mathbf{H}t}|1_{ex},\mu_{SC}\rangle \quad \text{and} \quad A_L(\vec{r},t) = {}_0\langle L^{(\ell)}(z)|e^{-\frac{i}{\hbar}\mathbf{H}t}|1_{ex},\mu_{SC}\rangle, \tag{S24}$$

In addition, the probability amplitude of finding the electron is in the excited state of QD is given as

$$A_{QD}(0,t) = {}_0\langle QD|e^{-\frac{i}{\hbar}\mathbf{H}t}|QD\rangle_0. \tag{S25}$$

Prove :

For the tilted dispersion relation of SWCNT, it is discussed by a curvature effect tight-binding Hamiltonian of an electron in terms of a two-by-two matrix in the $\vec{k}$ space:

$$\mathbf{H}_{SC} = \sum_{\vec{k}} \begin{pmatrix} \hat{c}^\dagger_{c,\vec{k}} & \hat{c}^\dagger_{v,\vec{k}} \end{pmatrix} \mathcal{H}_0 \begin{pmatrix} \hat{c}_{c,\vec{k}} \\ \hat{c}_{v,\vec{k}} \end{pmatrix}, \tag{S26}$$

Following curvature-induced tilting of the linear band Hamiltonian, which gives the asymmetric velocities, the Hamiltonian $\mathcal{H}_0(\vec{k})$ is given as

$$\mathcal{H}_0 = \sum_{\mu=0,1,2} \hbar\vec{v}_\mu\sigma^\mu\cdot\vec{k} + A_\mu^{(pse)}\sigma^\mu. \tag{S27}$$

Then $\hat{\mathbf{v}}_z = \frac{i}{\hbar}[\mathbf{H}_{SC},z]$ is given as

$$\hat{\mathbf{v}}_z = \frac{i}{\hbar}[\mathbf{H}_{SC},z] = \sum_{\mu=0,1,2}\sum_{\vec{k}} \begin{pmatrix} \hat{c}^\dagger_{c,\vec{k}} & \hat{c}^\dagger_{v,\vec{k}} \end{pmatrix} v_{z,\mu}\sigma^\mu \begin{pmatrix} \hat{c}_{c,\vec{k}} \\ \hat{c}_{v,\vec{k}} \end{pmatrix} \tag{S28}$$

or

$$\hat{\mathbf{v}}_z = \sum_{\vec{k}} \begin{pmatrix} \hat{c}^\dagger_{c,\vec{k}} & \hat{c}^\dagger_{v,\vec{k}} \end{pmatrix} \begin{pmatrix} \tau v_{z,0} & v_{z,1} - i\tau v_{z,2} \\ v_{z,1} + i\tau v_{z,2} & \tau v_{z,0} \end{pmatrix} \begin{pmatrix} \hat{c}_{c,\vec{k}} \\ \hat{c}_{v,\vec{k}} \end{pmatrix} \tag{S29}$$

Thus the conventional current density usually is obtained as

$$J_\eta^{(K)}(z,t) = {}_\eta\langle\Phi(z,t)|\hat{\mathbf{v}}_z|\Phi(z,t)\rangle_\eta = {}_0\langle\eta(z)|\hat{\mathbf{v}}_z|\eta(z)\rangle_0|A_\eta(z,t)|^2 = \eta v_\eta P_\eta(z,t) \tag{S30}$$

When the right- and left-going electron field operators are defined respectively in the form as

$$\hat{R}(z) = \sqrt{\frac{L}{2\pi}}\int_0^\infty dq\ e^{-iqz}\hat{c}_{c,q}|\chi_c(q)\rangle \ \text{ and } \ \hat{L}(z) = \sqrt{\frac{L}{2\pi}}\int_{-\infty}^0 dq\ e^{-iqz}\hat{c}_{c,q}|\chi_c(q)\rangle \tag{S31}$$

and

$$|R(z)\rangle_0 = \hat{R}(z)|0_{ph},\mu_{SC}\rangle \quad \text{and} \quad |L(z)\rangle_0 = \hat{L}(z)|0_{ph},\mu_{SC}\rangle \tag{S32}$$

Prove :

$$v_R =_0 \langle R(z)|\hat{\mathbf{v}}_z|R(z)\rangle_0 \quad \text{and} \quad v_L = -_0\langle L(z)|\hat{\mathbf{v}}_z|L(z)\rangle_0 \tag{S33}$$

Using Eq.(S32), Eq.(S31) and Eq.(13), we can write Eq.(S33) as

$$v_R = \frac{L}{2\pi}\int_0^\infty dq \int_0^\infty dq'\; e^{-i(q-q')z}\langle\mu_{SC}|\begin{pmatrix} e^{i\Phi_c(q)} & 1\end{pmatrix}\hat{c}^\dagger_{c,q}\hat{\mathbf{v}}_z\hat{c}_{c,q'}\begin{pmatrix} e^{i\Phi_c(q')} \\ 1\end{pmatrix}|\mu_{SC}\rangle \tag{S34}$$

and

$$v_L = -\frac{L}{2\pi}\int_{-\infty}^0 dq \int_{-\infty}^0 dq'\; e^{-i(q-q')z}\langle\mu_{SC}|\begin{pmatrix} e^{i\Phi_c(q)} & 1\end{pmatrix}\hat{c}^\dagger_{c,q}\hat{\mathbf{v}}_z\hat{c}_{c,q'}\begin{pmatrix} e^{i\Phi_c(q')} \\ 1\end{pmatrix}|\mu_{SC}\rangle \tag{S35}$$

Inserting Eq.(S29) into Eq.(S34) and Eq.(S35), we obtain

$$v_R = \frac{L}{4\pi}\int_0^\infty dq\, \left(2\tau v_{z,0} + e^{i\Phi_c(q)}(v_{z,1} - i\tau v_{z,2}) + e^{-i\Phi_c(q)}(v_{z,1} + i\tau v_{z,2})\right) \tag{S36}$$

and

$$v_L = -\frac{L}{4\pi}\int_{-\infty}^0 dq\, \left(2\tau v_{z,0} - e^{i\Phi_c(q)}(v_{z,1} - i\tau v_{z,2}) - e^{-i\Phi_c(q)}(v_{z,1} + i\tau v_{z,2})\right) \tag{S37}$$

Using Eq.(S14), the phase factor of spinor for $k_\phi = 0$,

$$e^{i\Phi_c(\vec{k})} = \frac{(v_{z,1} + i\tau v_{z,2})}{\sqrt{v_{z,1}^2 + v_{z,2}^2}}, \quad \text{and} \quad e^{-i\Phi_c(\vec{k})} = \frac{(v_{z,1} - i\tau v_{z,2})}{\sqrt{v_{z,1}^2 + v_{z,2}^2}}, \tag{S38}$$

we can write Eq.(S36) and Eq.(S37) as

$$v_R = (\frac{L}{4\pi})(\frac{2\pi}{L})\left(2\tau v_{z,0} + 2\sqrt{v_{z,1}^2 + v_{z,2}^2}\right) = \left(\sqrt{v_{z,1}^2 + v_{z,2}^2} + \tau v_{z,0}\right) \tag{S39}$$

and

$$v_L = -(\frac{L}{4\pi})(\frac{2\pi}{L})\left(2\tau v_{z,0} - 2\sqrt{v_{z,1}^2 + v_{z,2}^2}\right) = \left(\sqrt{v_{z,1}^2 + v_{z,2}^2} - \tau v_{z,0}\right) \tag{S40}$$

3. CONTINUITY EQUATION

The continuity equation for the probability density is written as the divergence theorem to obtain

$$\sum_{\eta=L,R}\left(\frac{\partial}{\partial z}J^{(K)}_\eta(z,t) + \frac{\partial}{\partial t}P_\eta(z,t)\right) = \sigma(t)\delta(z), \tag{S41}$$

where $\sigma(t)$ is the generation of the probability for electron transferred form QD to SWCNT per unit time, referred to as a "sources." being in the form that

$$\sigma(t) = \frac{g_f}{\hbar}\sum_{\eta=L,R} 2\,Im\left[A^*_\eta(0,t)A_{QD}(t)\right] + \Gamma P_{QD}(t) + \frac{\partial}{\partial t}P_{QD}(t). \tag{S42}$$

Prove :

The schrodinger equation of the dynamics in the full system is

$$\mathbf{H}|\Psi(t)\rangle = \left(\mathbf{H}_{SC} + \mathbf{H}_{QD} + \mathbf{H}_{SC-QD}\right)|\Psi(t)\rangle = i\hbar\frac{\partial}{\partial t}|\Psi(t)\rangle \tag{S43}$$

Therefore, the hermitian conjugate of this schrodinger equation is

$$\left(\mathbf{H}|\Psi(t)\rangle\right)^\dagger = \left(\left(\mathbf{H}_{SC} + \mathbf{H}_{QD} + \mathbf{H}_{SC-QD}\right)|\Psi(t)\rangle\right)^\dagger = -i\hbar\frac{\partial}{\partial t}\langle\Psi(t)| \tag{S44}$$

The inner products from Eq.(S13) and Eq.(S14) through $\langle\Psi(t)|$ and $|\Psi(t)\rangle$,respectively are obtained

$$\langle\Psi(t)|\mathbf{H}|\Psi(t)\rangle = \langle\Psi(t)|\left(\mathbf{H}_{SC} + \mathbf{H}_{QD} + \mathbf{H}_{SC-QD}\right)|\Psi(t)\rangle = i\hbar\langle\Psi(t)|\frac{\partial}{\partial t}|\Psi(t)\rangle \tag{S45}$$

and

$$(\mathbf{H}|\Psi(t)\rangle)^{\dagger}|\Psi(t)\rangle = \langle\Psi(t)|\left(\left(\mathbf{H}_{SC}+\mathbf{H}_{QD}+\mathbf{H}_{SC-QD}\right)|\Psi(t)\rangle\right)^{\dagger} = -i\hbar\left(\frac{\partial}{\partial t}\langle\Psi(t)|\right)|\Psi(t)\rangle. \tag{S46}$$

The dynamics of electrons by separating into three composition as the right or left-going state and occupation in the excited state of QD is

$$|\Psi(t)\rangle = \int dz\Big[A_R(z,t)|R(z)\rangle_0 + A_L(z,t)|L(z)\rangle_0\Big] + A_{QD}(t)|QD,1_{ex}\rangle_0. \tag{S47}$$

As we insert the state $|\Psi(t)\rangle$ in Eq.(S17) and Eq.(S15), the inner products with hermitian conjugate partners are

$$\langle\Psi(t)|\mathbf{H}_{SC}|\Psi(t)\rangle = \sum_{\eta=R,L}\int dz\int dz'\ A^*_\eta(z,t)A_\eta(z',t)\left[\langle\eta(z)|\sum_{\vec{k}}\begin{pmatrix}\hat{c}^\dagger_{c,\vec{k}} & \hat{c}^\dagger_{v,\vec{k}}\end{pmatrix}\mathcal{H}_0\begin{pmatrix}\hat{c}_{c,\vec{k}}\\ \hat{c}_{v,\vec{k}}\end{pmatrix}|\eta(z')\rangle\right] \tag{S48}$$

Following the define the measured state which find an electron moving as right-going $(z>0)$ or left-going $(z<0)$ in the conduction band with the band index $\ell=0$ (low energy conduction band) are

$$|R(z)\rangle_0 = \hat{R}^\dagger(z)|0_{ex},\mu_{SC}\rangle \text{ and } |L(z)\rangle_0 = \hat{L}^\dagger(z)|0_{ex},\mu_{SC}\rangle \text{ and } |QD,1_{ex}\rangle_0 = |1_{ex},\mu_{SC}\rangle\ , \tag{S49}$$

and the right- and left-going electron field operators are defined respectively in the form as

$$\hat{R}(z) = \sqrt{\frac{L}{2\pi}}\int_0^\infty dq\ e^{-iqz}\hat{c}_{c,q}|\chi_c(q)\rangle \text{ and } \hat{L}(z) = \sqrt{\frac{L}{2\pi}}\int_{-\infty}^0 dq\ e^{-iqz}\hat{c}_{c,q}|\chi_c(q)\rangle. \tag{S50}$$

As we insert Eq.(S19) and Eq.(S20) in Eq. (S18), we obtain as

$$\langle\Psi(t)|\mathbf{H}_{SC}|\Psi(t)\rangle = \frac{1}{2}\sum_{\mu=0,1,2}\int dz\ i\hbar\frac{\partial}{\partial z}\left[\langle R(z)|\vec{v}_\mu\sigma^\mu|R(z)\rangle P_R(z,t) + \langle L(z)|\vec{v}_\mu\sigma^\mu|L(z)\rangle P_L(z,t)\right] \tag{S51}$$

and

$$(\mathbf{H}_{SC}|\Psi(t)\rangle)^\dagger|\Psi(t)\rangle = \frac{1}{2}\sum_{\mu=0,1,2}\int dz(-i)\hbar\frac{\partial}{\partial z}\left[\langle R(z)|\vec{v}_\mu\sigma^\mu|R(z)\rangle P_R(z,t) + \langle L(z)|\vec{v}_\mu\sigma^\mu|L(z)\rangle P_L(z,t)\right] \tag{S52}$$

and

$$\langle\Psi(t)|\mathbf{H}_{SC-QD}|\Psi(t)\rangle = g_f\sum_{\eta=R,L}\int dz'\int dz\delta(z)\left[\langle\eta(z')|\hat{\eta}^\dagger(z)A^*_\eta(z',t)A_{QD}(0,t)\hat{a}_{ex}|QD,1_{ex}\rangle_0\right] \tag{S53}$$

and

$$(\mathbf{H}_{SC-QD}|\Psi(t)\rangle)^\dagger|\Psi(t)\rangle = g_f\sum_{\eta=R,L}\int dz'\int dz\delta(z)\left[\langle QD,1_{ex}|\hat{a}^\dagger_{ex}A^*_{QD}(0,t)A_\eta(z',t)\hat{\eta}(z)|\eta(z')\rangle\right] \tag{S54}$$

and

$$\langle\Psi(t)|\mathbf{H}_{QD}|\Psi(t)\rangle = \hbar(\omega_{ex}-i\Gamma/2)\langle QD,1_{ex}|A^*_{QD}(z,t)\hat{a}^\dagger_{ex}\hat{a}_{ex}A_{QD}(z,t)|QD,1_{ex}\rangle \tag{S55}$$

and

$$(\mathbf{H}_{QD}|\Psi(t)\rangle)^\dagger|\Psi(t)\rangle = \hbar(\omega_{ex}+i\Gamma/2)\langle QD,1_{ex}|A^*_{QD}(z,t)\hat{a}^\dagger_{ex}\hat{a}_{ex}A_{QD}(z,t)|QD,1_{ex}\rangle \tag{S56}$$

Subsequently, the difference of the inner products Eq.(S15) and Eq.(S16) is

$$\langle\Psi(t)|\mathbf{H}|\Psi(t)\rangle - (\mathbf{H}|\Psi(t)\rangle)^\dagger|\Psi(t)\rangle = i\hbar\frac{\partial}{\partial t}\langle\Psi(t)|\Psi(t)\rangle \tag{S57}$$

The right-hand side of Eq.(S24) can be represented by the composition of the right and left-going states with the occupation state in QD as

$$i\hbar\frac{\partial}{\partial t}\langle\Psi(t)|\Psi(t)\rangle = i\hbar\frac{\partial}{\partial t}\int dz\,(P_R(z,t)+P_L(z,t)) + i\hbar\frac{\partial}{\partial t}P_{QD}(z,t) \tag{S58}$$

In the same way, if we insert the inner products from Eq.(S18) to Eq.(S23) for the left-hand side of Eq.(S24), it represents the composition of the three component as

$$\langle \Psi(t)|\mathbf{H}|\Psi(t)\rangle - (\mathbf{H}|\Psi(t)\rangle)^{\dagger}|\Psi(t)\rangle =$$

$$\int dz \left(i\hbar \frac{\partial}{\partial z} \left[v_R P_R(z,t) - v_L P_L(z,t) \rangle \right] - i\hbar \Gamma P_{QD}(z,t) + i g_f \delta(z) \sum_{\eta=R,L} 2\, Im \left[A_\eta(z,t) A^*_{QD}(z,t) \right] \right) \tag{S59}$$

Eq.(25) equal to Eq.(26). As we divide these equations by $i\hbar$, the equation is

$$\int dz \left(\sum_{\eta=L,R} \frac{\partial}{\partial z} J^{(K)}_\eta(z,t) + \frac{\partial}{\partial t} P_\eta(z,t) - \sigma(t)\delta(z) \right) = 0, \tag{S60}$$

where $\sigma(t)$ is the generation of the probability for electron transferred form QD to SWCNT per unit time, or referred to as a "sources" being in the form

$$\sigma(t) = -\frac{g_f}{\hbar} \sum_{\eta=L,R} 2\, Im \left[A_\eta(0,t) A^*_{QD}(t) \right] + \Gamma P_{QD}(t) + \frac{\partial}{\partial t} P_{QD}(t). \tag{S61}$$

or

$$\sigma(t) = \frac{g_f}{\hbar} \sum_{\eta=L,R} 2\, Im \left[A^*_\eta(0,t) A_{QD}(t) \right] + \Gamma P_{QD}(t) + \frac{\partial}{\partial t} P_{QD}(t). \tag{S62}$$

Through Eq.(S27), we can reasonably calculate the current density and probabilty density further.

$$J^{(K)}_t(z,t) = J^{(K)}_R(z,t) + J^{(K)}_L(z,t) = v_R P_R(z,t) - v_L P_L(z,t) \tag{S63}$$

Consequently, the continuity equation for the probability density is written as the divergence theorem that

$$\sum_{\eta=L,R} \left(\frac{\partial}{\partial z} J^{(K)}_\eta(z,t) + \frac{\partial}{\partial t} P_\eta(z,t) \right) = \sigma(t)\delta(z), \tag{S64}$$
